# Genetical genomics: use all data

**DOI:** 10.1186/1471-2164-8-69

**Published:** 2007-03-12

**Authors:** Miguel Pérez-Enciso, José R Quevedo, Antonio Bahamonde

**Affiliations:** 1 Departament of Food and Animal Science, Veterinary School, Universitat Autònoma de Barcelona, 08193 Bellaterra, Spain; 2 Institut Català de Recerca i Estudis Avançats, 08010 Barcelona, Spain; 3 Artificial Intelligence Center, University of Oviedo at Gijón, 33271 Gijón, Spain

## Abstract

**Background:**

Genetical genomics is a very powerful tool to elucidate the basis of complex traits and disease susceptibility. Despite its relevance, however, statistical modeling of expression quantitative trait loci (eQTL) has not received the attention it deserves. Based on two reasonable assertions (i) a good model should consider all available variables as potential effects, and (ii) gene expressions are highly interconnected, we suggest that an eQTL model should consider the rest of expression levels as potential regressors, in addition to the markers.

**Results:**

It is shown that power can be increased with this strategy. We also show, using classical statistical and support vector machines techniques in a reanalysis of public data, that the external transcripts, i.e., transcripts other than the one being analysed, explain on average much more variability than the markers themselves. The presence of eQTL hotspots is reassessed in the light of these results.

**Conclusion:**

Model choice is a critical yet neglected issue in genetical genomics studies. Although we are far from having a general strategy for model choice in this area, we can at least propose that any transcript level is scanned not only for the markers genotyped but also for the rest of gene expression levels. Some sort of stepwise regression strategy can be used to select the final model.

## Background

Genetical genomics is currently a very active area of research, promising to improve dramatically our knowledge on the genetic architecture of complex traits, including disease susceptibility. Its goal is to identify the polymorphisms responsible for the variation in gene expression levels and thus to improve our understanding of how gene networks are organised in an organism. Thus far, genetical genomics experiments have been analysed considering each expression level one at a time and using fairly simple statistical models, correcting only, e.g., by sex. As a consequence, the results are a collection of successive quantitative trait loci analysis (*eQTL *in the terminology introduced by Schadt et al. [1,2]), where each gene expression level is analysed independently. It is surprising that much effort has been dedicated to issues like data normalization [3] or computing efficiency [4] whereas modelling the trait itself (i.e., the expression level) has been severely neglected.

**Figure 1:**
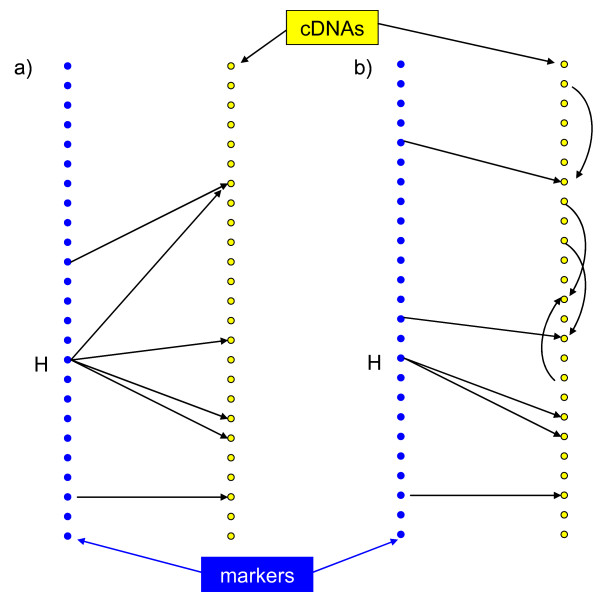
**Approach proposed in this paper**. Schematic representation of the analysis of genetical genomics experiment, the blue circles represent the markers tested and the yellow circles, each of the transcript levels analysed, an arrow signifies that the effect has been included in the model for the transcript. The left cartoon (**a**) represents the current strategy for eQTL searching: it consists of including the most significant marker in the model when testing each transcript independently. Several arrows pointing to a transcript means that the transcript is affected by several QTL, while many arrows starting in a single marker represents an eQTL hotspot (**H**). The right cartoon (**b**) presents the strategy proposed here, which suggests that external expression levels can be included as covariates in the model for the expression level studied (the arrows that start and end at the cDNA circles). Including cDNAs in the model can dramatically affect the final eQTL map, some positions may be shifted, some previous eQTL may disappear or some new appear. The bottom line of this approach is that all markers and all expression levels are potential regressors to be considered. The optimum model could be chosen using some of the available criteria, like AIC, BIC, DIC or AUC among others.

Based on two rather reasonable assertions (i) a good modelling strategy should consider all available variables as potential effects in the model, and (ii) gene expressions are highly interconnected, we suggest that an eQTL model for a given gene should consider the rest of expression levels as potential regressors as well as the markers to identify regulatory polymorphisms. The current models are rather naive, which may lead to spurious results, whereas high intercorrelation between transcript levels can make QTL signals difficult to interpret. For instance, it may not be possible to disentangle between a direct effect of the marker on the gene or an indirect (spurious) effect caused by an intermediate gene in the same or co-regulated metabolic route. Here we propose a new analysis paradigm, depicted in Figure 1, whereby for each trait (i.e., any given expression level), all remaining cDNA levels are potential regressors that can be included in the model.

The proposed approach provides new insight into genetical genomics data, as we argue later, but first the researcher should be aware of the interpretation of the different strategies. In the usual modelling strategy, when only markers are fitted to explain the expression level, one is interested in picking up markers associated to the trait *regardless *of other effects. If markers and additional correlated expression levels are included in the model, one will select only those markers that are *conditionally *associated to the phenotype of interest, that is, after removing the direct effect of external expression levels on the phenotype. The rationale for this is to avoid confounding and improve power by reducing environmental noise. In practice, the issue of what covariables to include can be a difficult choice. Suppose that we are analyzing the effect of some polymorphisms on a phenotype in human populations. Suppose also that this phenotype is affected by sex. If one includes height as well as sex in the model, and because height and sex are correlated, it might be that a 'true' marker effect is attenuated. Here, fitting the marker within each sex class (i.e., fitting an interaction sex × marker) could improve model performance.

A relevant question in our approach is: what is the relative importance of each set of variables, discrete (markers) vs. continuous (transcript levels). This question is equivalent to disentangling the relevance of genetics vs. environment because transcript levels are part of the 'environment'. There are two broad approaches in the literature to measure the relevance of variables based, respectively, on statistical and on artificial intelligence techniques. The former is based on fitting two competing models, with and without the variable of interest; a typical measure of variable importance is the P-value obtained in, say, a likelihood ratio test. The P-value measures essentially the probability of having obtained the data when the null model is true, the smaller this probability is, the less likely the null model holds. In contrast to statistical methodologies, artificial intelligence techniques are not too popular in genetics yet. Among the pleiade of artificial intelligence techniques, support vector machines (SVM) have emerged as one of the most reliable and efficient methodologies. SVM are a powerful family of algorithms for learning classification and regression tasks [5]. They are based on the minimization of the structural risk of errors by means of well-known and well-founded techniques of quadratic programming. Using the so-called kernel trick, SVM can learn linear and nonlinear functions from either continuous or discrete variables. An important feature of SVM is that they can successfully handle datasets with a small number of observations and thousands of independent variables, the so called 'large p small n paradigm', which make them an attractive tool for microarray data [6-8] or for information retrieval where each document is described by a vector with as many indexes as possible words [9]. Importantly, SVM can be endowed with algorithms [10,11] that provide an ordered list of variables according to their relevance in a prediction task. However, and in contrast to classical statistical methods, the relevance of each individual variable can not be quantified with these algorithms.

In this work we explore the consequences of model choice in eQTL studies reanalyzing public data with maximum likelihood and SVM techniques. We show that, in fact, most of the variation observed in gene expression is largely explained by external expression levels, while the influence of polymorphic markers (the eQTL itself) is limited. We show that this can have a dramatic influence on the results obtained. Figure 1 illustrates the point made in this article.

## Results and discussion

### Variable relevance

**Figure 2:**
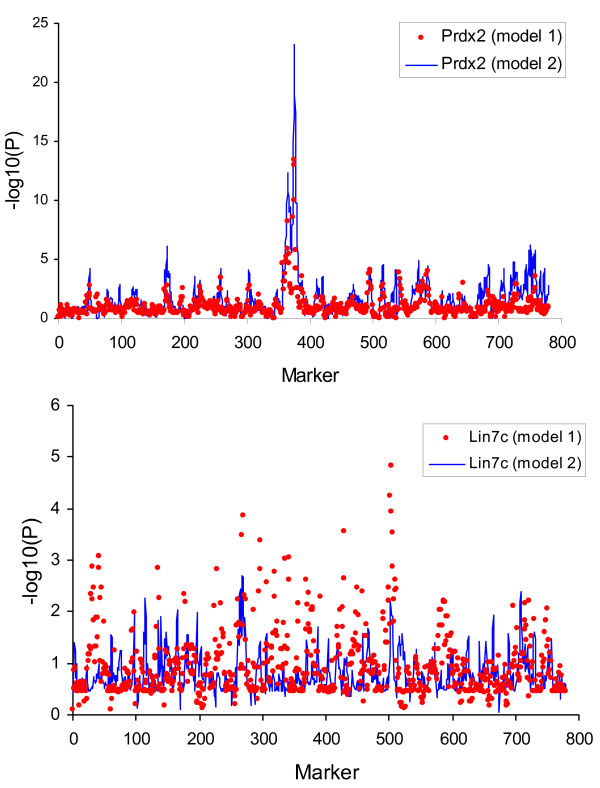
**Comparison of eQTL profiles**. P-value Profiles with model 1 (red dots) or 2 (blue line) for two genes, *Prdx2 *(top) and *Lin7c *(bottom). P-values are in log10 scale. Model 1 considers only the marker in the model, whereas model 2 also includes the most associated transcript level.

Throughout this work, we compared the results for eQTL scan with two models, a first model (model 1) where the only effects are a general mean and the marker, and a second model (model 2) where external cDNA levels were also included as covariate (see methods). Chesler et al. [12] listed a series of ~70 highly significant eQTL (genome wise P-values < 0.04) using a model 1 type strategy. One of the most significant QTL affected the expression of the gene *peroxiredoxin *2 (*Prdx2*), which plays a major role in protecting against oxidative stress. Figure 2 (top) shows the results with model (1), i.e., the classical approach. As expected, we found a highly significant QTL (nominal P-value ~10^-15^), in agreement with published results. However, when we scanned for association not only the 779 markers genotyped but also the rest of cDNA levels, the most associated variable was actually the transcript level of gene *Psma7 *(P < 10^-20^). Interestingly, this gene is involved in regulating the hypoxia-inducible factor-1alpha, a transcription factor important for cellular responses to oxygen tension. Next, we included *Psma7 *level as covariate in the eQTL model for *Prdx2 *and we reanalysed the data with model (2), testing as before for the associated significance of each marker in turn. The results are also in Figure 2 (top). Although the profile is similar, the QTL is now far more significant (P-value = 10^-23 ^now *vs. *10^-15 ^in the previous model). This observation strongly suggests that *Psma7 *plays an important role in modifying the expression of *Prdx2*, but also that its influence is purely environmental and probably under different genetic control; as a result, including *Psma7 *in the model removes a large part of the residual variation.

The opposite phenomenon was observed with the transcript level of *Lin7c*, one of the most highly connected gene in the brain [12]. In this case, the original eQTL had a nominal P-value ~10^-5 ^with the usual model (model 1). This significance decreased when *Sacm1l *expression level was included using model (2) (P = 2 × 10^-3^). *Sacm1l *transcript level was the most significant factor associated with *Lin7c *expression (P < 10^-53^), i.e., much more significant than any marker. It should also be noted that the position of the maximum statistics was shifted, from marker *D12Mit234 *in chromosome 12 to *D6Mit116 *in chromosome 6. Interestingly, the expression level of *Sacm1l *had an eQTL also in the neighborhood of marker *D12Mit234 *with model 1. This likely occurs because both traits are highly correlated (ρ = 0.99). Schadt et al. [2] proposed to compare likelihoods P(x_1_|m) and P(x_1_|x_2_), where x_1 _and x_2 _are cDNA measures and m, marker genotypes, in order to disentangle whether m or x_2 _are causal to x_1_. Here, we compared P(x_*Lin7c *_| x_*Sacm1l*_) vs. P(x_*Sacm1l *_| x_*Lin7c*_) but they were almost identical and thus we cannot resolve whether one gene is causal to the other by using only statistical evidence, whereas P(x_*Lin7c *_| *D12Mit234*) was slightly more significant (P = 10^-5^) than P(x_*Sacm1l *_| *D12Mit234*), P = 10^-3^. All this hints that *Lin7c *and *Sacm1l *are mediated through the same causal effects but that it seems that their association with D12Mit234 could be actually a false positive because it is far less significant than other eQTL found in this same study.

**Figure 3:**
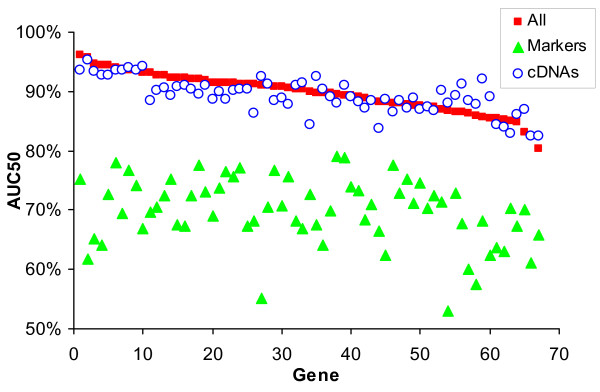
**AUCs for gene expression levels**. Comparison between AUC for 67 gene expression levels considering the best 50 predictive variables chosen among all markers and cDNA levels (red solid squares), the best 50 variables chosen among all markers (green solid triangles) and considering the best 50 variables chosen among all transcript levels (blue open circles). All three AUCs for each expression level are in the same abscissa's position, genes were ranked according to AUC using all variables. It can be seen that using only markers results in consistently lower AUC, whereas there are no large differences between using all variables or only transcript levels. For some genes (23 out of 67), AUCs using only cDNAs were slightly better than using all variables, this occurred because the RFE algorithm [10] may not completely remove redundant information from all variables and thus does not always guarantee the absolute maximum. The 67 genes shown were chosen within those with most significant QTLs in Chesler et al. (2005). Thus, one should expect that markers are better predictors, and consequently higher AUC, for these genes than for a random gene. Note that an AUC of 50% means than the criterion is no better than a random ordering.

Table 1 shows the associated P-levels and the goodness of fit statistics provided by SVM (AUC, see methods) of marker and expression levels for the ten most significant QTL reported in Chesler et al. [12]. The results corresponding to the most highly connected gene (*Lin7c*) are also presented. This Table illustrates well the relevance of external transcript levels as compared to markers. Note that the most associated variable was a transcript level rather than a marker in six out of ten genes, and this occurred for the most significant QTL, i.e., where the P-values for associated markers are most significant. Figure 3 displays the AUC obtained with the best 50 markers, the best 50 transcript levels, or the 50 best variables (including both markers and gene levels) in the 67 genes that exhibited the most significant QTL. Again, it can be clearly seen that transcript levels have a significantly and consistently better predictive ability than markers. For a random cDNA, we will expect that external transcript levels will be even more relevant than for those with highly significant QTL. In the case of the most highly connected gene, *Lin7c*, this is evident (Table 1). Thus, both classical statistics and SVM methods suggest that, on average, external transcript levels are better predictors of a given cDNA level than markers.

The above considerations should not imply that the most significant variable is *always *an external cDNA level for all genes, and thus that model (2) is to be preferred over model (1). We observed that usual model (1) was to be preferred in about 25% of the most significant QTL listed by Chesler et al[12]. In Table 1, the most significant variable was a marker rather than a transcript level in four out of ten genes (*Mela*, *Myoc*, *Cd59a*, and *Krtl-12*). We investigated whether modeling can nevertheless be improved in these cases. To do that, we searched the next most significant effect among all external cDNAs and the rest of markers, computing its P-value after fitting the QTL. We observed that the next most significant variable was a transcript which in turn was highly significant (P-values ranged 10^-8 ^– 10^-30^). Note that this is surely an underestimation of the influence because we were considering those expression levels for which a marker (QTL) is the most significant effect. The conclusion that we can draw, again, is that external cDNAs are likely to be very important factors to be considered in genetical genomics studies.

### Reanalyzing hotspots

The observation of eQTL hotspots, i.e., genome regions that seem to harbour a much higher number of QTL than expected by chance has been largely debated in the literature [1,13-15]. This is a remarkable observation and is tempting to look for a functional significance to these regions. It suggests the presence of key regulatory, polymorphic motifs in the genome that can have a profound influence on the genome transcription activity. In a previous simulation study we observed that QTL hotspots appeared even when genotypes and microarrays were shuffled, simply as a consequence of the high correlation that exists between many expression levels [16]. It is important to notice, though, that we can use the correlation between cDNA levels to our advantage in order to improve eQTL modelling dramatically.

To investigate the nature of eQTL hotspots and the influence of model choice, we chose nine genes that were reported by Chesler et al. [12] as influenced by the largest trans regulatory QTL hotspot, i.e., that close to marker *D6Mit150 *on murine chromosome 6. The nine genes chosen were *Reln*, *Chrng*, *Slc6a1*, *Calm4*, *Mapk6*, *Adra2b*, *Mapk1*, *Gad1*, and *Htr4*. As before, we fitted models (1) and (2). In all analyses with model (1), we found that the most significant marker was *D6Mit254*, the closest marker to *D6Mit150 *and also in chromosome 6, thus in agreement with published results. The P-values of the QTL with model (1) are in Table 2, they are in the order of 10^-3 ^– 10^-4^. Next, we scanned all transcript levels for each of the nine genes, the most significant ones for each gene are also listed in the Table. Note that the P-values are much smaller than the QTL (P-value < 10^-30 ^in all cases), which clearly shows that the nine expression levels studied are much more influenced by external transcript levels than by any of the polymorphisms genotyped. We also performed a QTL scan for these selected external transcripts and we found that they mapped to regions distinct from chromosome 6, and thus that they were not members of the QTL hotspot (results not presented). Finally, we included the relevant transcript as covariate for each of the nine genes and we repeated the QTL analysis (model 2). Results are also in Table 2 (last two columns).

Two aspects are important. First, the QTL were more significant now than with model (1); sometimes significance increased dramatically, e.g., P-value changed from 10^-4 ^to 10^-21 ^for gene *Reln*. This is a clear evidence that expression levels included in the model remove noise and thus may increase power for QTL detection, as we observed previously (Figure 2 top). We did not always observe this, in other instances we found that adjusting for transcript levels decreased QTL significance (Figure 2 bottom). In these latter cases we can conclude that the QTL found with the simple model is an artefact and that the QTL was truly affecting other gene expression level. The second noticeable aspect in Table 2 is that the QTL location was shifted for some transcripts (*Reln*, *Calm4*, *Adra2b*, and *Htr4*). Although we did not systematically search for all cDNA levels affected by this QTL, it is clear that the number of the genes in the hotspot has been reduced by ~50%. This is a challenging result and calls for revisiting the significance of eQTL hotspots, as they are highly dependent on the model used. From a purely statistical – rational – point of view, a model that includes a highly correlated transcript level is to be preferred over one that includes only the marker: P-values in the order of 10^-30 ^to 10^-45 ^*vs*. ~10^-4^.

## Conclusion

Including external expression levels in the model can improve statistical inference, decreasing the rate of false positives and increasing power (e.g., Figure 2). This is a consequence of the biological fact that regulation of gene expression is highly interconnected, resulting in the complex intercorrelations that exist in microarray data. Seemingly, a high fraction of this correlation is purely environmental. In fact, as Figure 3 suggests, expression levels are far more important than markers in explaining the observed variability. In other words, the variation in a given expression level is more likely to be affected by the expression levels of related genes than directly caused by marker polymorphisms. Thus, we can argue that expression levels behave as noisy environmental factors, very much like say age, sex or batch in a regular statistical analysis. But in all likelihood, each expression level will behave differently and thus we will require specific models for each expression level. Thus, automated and efficient modeling strategies are badly needed if we are to exploit all information contained in genetical genomics studies.

In conclusion, model choice is a critical yet neglected issue in genetical genomics studies. Although we are far from having a general strategy for model choice in this area, we can at least propose that any transcript level is scanned not only for the markers genotyped but also for the rest of gene expression levels. Some sort of stepwise regression strategy can be used to select the final model. This will illuminate what a QTL hotspot is really made of and will improve our ability to reconstruct genetic networks from genetical genomics experiments.

## Methods

### Data

Chesler et al.'s [12] experiment consists of a set of 35 BxD mouse recombinant inbred lines. Brain tissue from 100 pools of individuals were arrayed with Affymetrix U74Av2 arrays chips and a panel of 779 markers was genotyped. Each array experiment was made up with a pool of brain tissue (excluding olfactory bulb, retina or neurohypohysis) from three individuals of the same sex. The data set was downloaded from the GeneNetwork site [17].

### Maximum likelihood techniques

Two main models were used to reanalyze the data. In model (1), the j-th expression level for i-th individual (i = 1, n) is modelled as

yij=μ+∑tλit gt+εij,     (1)
 MathType@MTEF@5@5@+=feaafiart1ev1aaatCvAUfKttLearuWrP9MDH5MBPbIqV92AaeXatLxBI9gBaebbnrfifHhDYfgasaacH8akY=wiFfYdH8Gipec8Eeeu0xXdbba9frFj0=OqFfea0dXdd9vqai=hGuQ8kuc9pgc9s8qqaq=dirpe0xb9q8qiLsFr0=vr0=vr0dc8meaabaqaciaacaGaaeqabaqabeGadaaakeaacqqG5bqEdaWgaaWcbaGaeeyAaKMaeeOAaOgabeaakiabg2da9iabeY7aTjabgUcaRmaaqafabaGaeq4UdW2aaSbaaSqaaiabdMgaPjabdsha0bqabaGccqqGGaaicqqGNbWzdaWgaaWcbaGaeeiDaqhabeaaaeaacqqG0baDaeqaniabggHiLdGccqGHRaWkcqaH1oqzdaWgaaWcbaGaeeyAaKMaeeOAaOgabeaakiabcYcaSiaaxMaacaWLjaWaaeWaaeaacqaIXaqmaiaawIcacaGLPaaaaaa@4ABB@

where μ is the general mean or any other fixed effects that may be included in the model, λ is a 0/1 indicator variable that identifies the genotype of the individual for the marker considered (i.e., λ_it _= 1 if the i-th individual has genotype t, 0 otherwise), and ε is the residual. Note that we assume that marker density is very high so that we test each individual marker in turn instead of carrying out a QTL scan. This is done here for simplicity and computational speed as is straight forward to generalize (1) to other situations. Carlborg et al. [18] found no large differences between single marker and interval mapping in this context. In model 2 we allow, in addition, that cDNAs other than the one analysed can be included in the model as covariates, i.e.,

yij=μ+∑tλit gt+∑k≠jδkβk yik+εij     (2)
 MathType@MTEF@5@5@+=feaafiart1ev1aaatCvAUfKttLearuWrP9MDH5MBPbIqV92AaeXatLxBI9gBaebbnrfifHhDYfgasaacH8akY=wiFfYdH8Gipec8Eeeu0xXdbba9frFj0=OqFfea0dXdd9vqai=hGuQ8kuc9pgc9s8qqaq=dirpe0xb9q8qiLsFr0=vr0=vr0dc8meaabaqaciaacaGaaeqabaqabeGadaaakeaacqqG5bqEdaWgaaWcbaGaeeyAaKMaeeOAaOgabeaakiabg2da9iabeY7aTjabgUcaRmaaqafabaGaeq4UdW2aaSbaaSqaaiabdMgaPjabdsha0bqabaGccqqGGaaicqqGNbWzdaWgaaWcbaGaeeiDaqhabeaaaeaacqqG0baDaeqaniabggHiLdGccqGHRaWkdaaeqbqaaiabes7aKnaaBaaaleaacqWGRbWAaeqaaOGaeqOSdi2aaSbaaSqaaiabdUgaRbqabaGccqqGGaaicqqG5bqEdaWgaaWcbaGaeeyAaKMaee4AaSgabeaakiabgUcaRiabew7aLnaaBaaaleaacqqGPbqAcqqGQbGAaeqaaaqaaiabbUgaRjabgcMi5kabbQgaQbqab0GaeyyeIuoakiaaxMaacaWLjaWaaeWaaeaacqaIYaGmaiaawIcacaGLPaaaaaa@5CF2@

where δ_k _is an indicator variable with value 1 if the cDNA level k is included in the model with covariate coefficient β_k_, 0 otherwise. Note that a most difficult problem can be choosing the adequate set of δ's. For this work, we scanned all cDNAs and we included in (2) only the most significant cDNA. Models (2) vs. (1) were compared with a likelihood ratio test, and P-values were computed assuming the usual Chi-squared approximation. Likelihood was maximized using an EM algorithm implemented in package Qxpak [19].

### Support vector machines techniques

SVM techniques are a well known tool for classification and prediction [20]. The rationale for using SVM in this context was to use an alternative to maximum likelihood to identify the variables that best predict the trait (expression level) of interest. As in the previous section, suppose we have an n-dimensional real vector containing the expression level to be studied (**y**), and a collection of d-dimensional real vectors **x**_i _that contains the descriptive variables (i.e, all markers and the rest of cDNAs). The goal of SVM is to produce a predictive function for the expressions levels of each individual. Therefore, the input of a SVM can be collected in a set of pairs **S **= {(**x**_1_', y_1_),...,(**x**_n_', y_n_)}, while the output is a vector w* and a scalar b* such that the function h defined by

h(**x**) = w*' **x **+ b*     (3)

is a prediction of the expression level y of an individual described by **x**. An important issue is to fix the criterion for measuring the quality of the prediction. In our case, the aim is to produce a function h (Eq. 3) such that the relative ordering of (h(**x**_1_),...,h(**x**_m_)) is as close as possible with the observed ordering of (y_1_,...,y_n_). For this purpose we used the loss function [21] that returns the number of pairs (i, j) whose predicted relative ordering (h(**x**_i_), h(**x**_j_)) is swapped with respect to its observed ordering of (y_i_, y_j_). Formally, the loss of h in the set is defined as the probability

ΔSP(h,S)=P(h(xi)≤h(xj)|yi>yj)=∑i,j:yi>yjI{h(xi)≤h(xj)}∑i,jI{yi>yj}     (4)
 MathType@MTEF@5@5@+=feaafiart1ev1aaatCvAUfKttLearuWrP9MDH5MBPbIqV92AaeXatLxBI9gBaebbnrfifHhDYfgasaacH8akY=wiFfYdH8Gipec8Eeeu0xXdbba9frFj0=OqFfea0dXdd9vqai=hGuQ8kuc9pgc9s8qqaq=dirpe0xb9q8qiLsFr0=vr0=vr0dc8meaabaqaciaacaGaaeqabaqabeGadaaakeaacqqHuoardaWgaaWcbaGaee4uamLaeeiuaafabeaakiabbIcaOiabbIgaOjabbYcaSiabbofatjabbMcaPiabg2da9iabbcfaqnaabmaabaGaeeiAaGMaeeikaGIaeCiEaG3aaSbaaSqaaiabbMgaPbqabaGccqqGPaqkcqGHKjYOcqqGObaAcqqGOaakcqWH4baEdaWgaaWcbaGaeeOAaOgabeaakiabbMcaPiabbYha8jabbMha5naaBaaaleaacqqGPbqAaeqaaOGaeyOpa4JaeeyEaK3aaSbaaSqaaiabbQgaQbqabaaakiaawIcacaGLPaaacqGH9aqpdaWcaaqaamaaqababaGaeeysaKKaee4EaSNaeeiAaGMaeeikaGIaeCiEaG3aaSbaaSqaaiabbMgaPbqabaGccqqGPaqkcqGHKjYOcqqGObaAcqqGOaakcqWH4baEdaWgaaWcbaGaeeOAaOgabeaakiabbMcaPiabb2ha9bWcbaGaeeyAaKMaeeilaWIaeeOAaOMaeeOoaOJaeeyEaK3aaSbaaWqaaiabbMgaPbqabaWccqGH+aGpcqqG5bqEdaWgaaadbaGaeeOAaOgabeaaaSqab0GaeyyeIuoaaOqaamaaqababaGaeeysaKKaee4EaSNaeeyEaK3aaSbaaSqaaiabbMgaPbqabaGccqGH+aGpcqqG5bqEdaWgaaWcbaGaeeOAaOgabeaakiabb2ha9bWcbaGaeeyAaKMaeeilaWIaeeOAaOgabeqdcqGHris5aaaakiaaxMaacaWLjaWaaeWaaeaacqaI0aanaiaawIcacaGLPaaaaaa@84F2@

where I{p(x)} is the function that returns 1 when the predicate p(x) is true, 0 otherwise. This loss function can be seen as a generalization of the complement of the Area Under a Receiver Operating Characteristic (ROC) curve, AUC for short. Hanley and McNeil [22] showed that the AUC is the probability of a correct ranking and thus AUC coincides with the value of the Wilcoxon-Mann-Whitney non parametric statistic. Here, we report

AUC = 100(1 - Δ_SP_(h, T))     (5)

as a measure of the goodness of the SVM prediction models. Technically, the SVM seeks w* and b* as the solution to the following convex quadratic optimization problem,

min12w'w+C∑i=1n(ξi−−ξi+)     (6)
 MathType@MTEF@5@5@+=feaafiart1ev1aaatCvAUfKttLearuWrP9MDH5MBPbIqV92AaeXatLxBI9gBaebbnrfifHhDYfgasaacH8akY=wiFfYdH8Gipec8Eeeu0xXdbba9frFj0=OqFfea0dXdd9vqai=hGuQ8kuc9pgc9s8qqaq=dirpe0xb9q8qiLsFr0=vr0=vr0dc8meaabaqaciaacaGaaeqabaqabeGadaaakeaacqqGTbqBcqqGPbqAcqqGUbGBdaWcaaqaaiabigdaXaqaaiabikdaYaaacqWH3bWDcqWHNaWjcqWH3bWDcqGHRaWkcqWGdbWqdaaeWbqaamaabmaabaGaeqOVdG3aa0baaSqaaiabdMgaPbqaaiabgkHiTaaakiabgkHiTiabe67a4naaDaaaleaacqWGPbqAaeaacqGHRaWkaaaakiaawIcacaGLPaaaaSqaaiabdMgaPjabg2da9iabigdaXaqaaiabd6gaUbqdcqGHris5aOGaaCzcaiaaxMaadaqadaqaaiabiAda2aGaayjkaiaawMcaaaaa@4E35@

subject to

(**w'x**_**i **_+ *b*) - *y*_*i *_≤ ∈ + ξi+
 MathType@MTEF@5@5@+=feaafiart1ev1aaatCvAUfKttLearuWrP9MDH5MBPbIqV92AaeXatLxBI9gBaebbnrfifHhDYfgasaacH8akY=wiFfYdH8Gipec8Eeeu0xXdbba9frFj0=OqFfea0dXdd9vqai=hGuQ8kuc9pgc9s8qqaq=dirpe0xb9q8qiLsFr0=vr0=vr0dc8meaabaqaciaacaGaaeqabaqabeGadaaakeaacqaH+oaEdaqhaaWcbaGaemyAaKgabaGaey4kaScaaaaa@30D9@ and *y*_*i *_- (**w'x**_i _+ *b*) ≤ ∈ + ξi−
 MathType@MTEF@5@5@+=feaafiart1ev1aaatCvAUfKttLearuWrP9MDH5MBPbIqV92AaeXatLxBI9gBaebbnrfifHhDYfgasaacH8akY=wiFfYdH8Gipec8Eeeu0xXdbba9frFj0=OqFfea0dXdd9vqai=hGuQ8kuc9pgc9s8qqaq=dirpe0xb9q8qiLsFr0=vr0=vr0dc8meaabaqaciaacaGaaeqabaqabeGadaaakeaacqaH+oaEdaqhaaWcbaGaemyAaKgabaGaeyOeI0caaaaa@30E4@,

where *C *is the regularization parameter and ξ are slack variables (ξ^+^, ξ^- ^≥ 0). Notice that the regression approach is similar to fitting the rank, although the function optimised by regression is not exactly a measure of the coherence between observed and predicted rankings. In fact, we could have chosen a SVM solution where the goal was to optimise the loss function AUC (4) directly, see e.g. Joachims [23,24]. However, the results achieved with regression were good enough and they are faster to obtain.

We used SVM^light ^software [9] to produce regressors that were evaluated with a 10 fold cross validation using the AUC loss function. This means that the whole dataset was randomly split into 10 partitions, each resulting in a training subset and a test subset. Equation (6) is used by SVM to produce a function h (Eq. 3) using the training subset, whereas the function h is evaluated (Eqns. 4 and 5) using the test subset. The performance estimation returned by the cross-validation method is the mean over all 10 partitions. The kernel used was linear, *C *was set to 1 (usually the default value in most SVM environments), and the parameter ∈ was set to 0.01, the default value in the implementation used.

The markers were dealt as discrete variables with each of the three values (the three genotypes) transformed into three Boolean attributes. Thus, when a marker was found to be among the 50 most relevant for a given trait, the three associated Boolean variables were included in the corresponding model. The algorithm used to discover relevancies was the so-called Recursive Feature Elimination (RFE) [10]; a simple yet efficient method when the kernel is linear. We run SVM for several cDNA levels considering as predictors either all variables (cDNAs and markers), only markers or only cDNA levels. We set a maximum of 50 variables to be included in the decission rule h.

## Abbreviations

AUC: Area under a receiver operating characteristic curve; QTL (eQTL): (expression) quantitative trait locus; RFE, Recursive Feature Elinitaion; SVM: support vector machine.

## Authors' contributions

MPE conceived the research, all performed research, MPE and AB wrote the paper. All authors read and approved the manuscript.

## Figures and Tables

**Table 1:** Associated P-values and AUC for some of the most significant QTL reported by Chesler et al.(2005)

	Best marker^a^			Best transcript^b^			AUC50(%) ^c^		
	
	Name	-log10 P-value^d^	AUC%	Name	-log 10 P-value^d^	AUC%	Marker	Transcript	All
	
Trans-QTL									
*Mela*	*D9Mit196*	23	72	*Cap1*	6	65	76	88	93
*Myoc*	*D2Mit237*	14	71	*Pam*	7	66	77	88	91
*Cd59a*	*D13Mit11*	12	72	*A08Rik*	11	57	74	89	89
*Myh9*	*D19Mit35*	5	61	*Igfbp5*	18	91	71	89	91
*Pitpnb*	*S14Gnf055.010*	6	63	*Rab7*	20	77	71	88	88
Cis-QTL									

*Prdx2*	*S08Gnf094.275*	15	71	*Psma7*	20	74	69	94	94
*Kcnj9*	*D9Mit11*	7	62	*Tnp1*	24	77	62	95	96
*Krt1-12*	*D11Mit58*	13	70	*Mrps7*	10	71	73	89	89
*Ntan1*	*D12Nyu7*	5	57	*K22Rik*	39	80	63	84	85
*Mrpl48*	*D7Mit17*	11	73	*Mcee*	23	67	77	90	91
Largest-clique									
*Lin7c*	*D12Mit84*	5	62	*Sacm1l*	53	90	80	97	97

a The marker shown is the most associated to the cDNA level of the gene in the first column.

^b ^The gene name shown is that whose cDNA level is most associated to the cDNA level of the gene in the first column.

^c ^AUC50 is the AUC obtained with the best 50 variables, the three columns refer to AUC obtained when only markers, only transcripts or all variables, respectively, are considered as predictors.

^d ^Values reported are -log_10 _(P-value), that is a value of x means that significance is 10^-x^.

**Table 2:** QTL results for a subset of genes pertaining to a QTL hotspot localised around marker *D6Mit254 *(chr. 6).

Gene	-log10 P-value of QTL (model 1) ^a,b^	Best transcript	P-value of best transcript^a^	Position of QTL (model 2)^c^	-log10 P-value of QTL (model 2) ^a,d^
*Reln*	4.4	*0610039D01Rik*	30.0	*S05Gnf018.190*	21.0
*Chrng*	4.1	*Gpx1*	32.3	*D6Mit254*	5.6
*Slc6a1*	3.7	*Mad2l1*	35.5	*D6Mit254*	5.9
*Calm4*	3.6	*Adprhl2*	37.8	*S17Gnf094.470*	7.2
*Mapk6*	3.5	*Tusc2*	41.7	*D6Mit254*	10.9
*Adra2b*	3.3	*Sox11*	30.9	*S04Gnf147.400*	3.6
*Mapk1*	3.3	*1110011K10Rik*	34.4	*D6Mit254*	6.2
*Gad1*	3.0	*Bzrp*	45.3	*D6Mit254*	3.2
*Htr4*	2.5	*Hbb-b2*	45.5	*D18Mit19*	11.7

^a ^Values reported are -log_10 _(P-value), that is a value of x means that significance is 10^-x^.

^b ^P-value when only the marker is included in the model.

^c ^QTL position when the best transcript is also included in the model.

^d ^QTL P-value when the best transcript is also included in the model.
